# Psychiatric hospital admission and later crime, mental health, and labor market outcomes

**DOI:** 10.1002/hec.4186

**Published:** 2020-11-02

**Authors:** Rasmus Landersø, Peter Fallesen

**Affiliations:** ^1^ Rockwool Foundation Research Copenhagen Denmark; ^2^ Swedish Institute for Social Research (SOFI) Stockholm University Stockholm Sweden

**Keywords:** crime, inpatient care, labor market, mental health

## Abstract

Most OECD countries have downsized treatment capacity at psychiatric hospitals substantially. We investigate consequences of these reductions by studying how the decision whether to admit individuals in mental distress to a psychiatric hospital affects their subsequent crime, treatment trajectories, and labor market outcomes. To circumvent nonrandom selection into admission, we use a proxy of occupancy rates prior to a patient's first contact with a psychiatric hospital as an instrument. We find that admissions reduce criminal behavior, likely due to incapacitation, and predominantly for males and those with a criminal record. Furthermore, admission lowers patients' subsequent labor market attachment, likely because a psychiatric hospital admission is an eligibility criterion for welfare benefits.

## 1 INTRODUCTION

Most OECD countries have downsized treatment capacity at psychiatric hospitals substantially during the past decades (WHO, 2011a), and psychiatric health care is increasingly provided as outpatient treatment (e.g., Oosterhuis, 2005). This potentially leads to capacity constraints in immediate hospital care. If patients' immediate needs go unaddressed, it could result in large externalities and indirect costs for society, as individuals with psychiatric disorders suffer from a host of problems (see e.g., Ettner, Frank, & Kessler, 1997; Greve & Nielsen, 2013; Kupers & Toch, 1999). Yet, the consequences of lowering hospital admission rates are at present largely unknown.

This study examines the social and economic effects of admitting an individual to inpatient treatment at a psychiatric hospital. We use administrative data for the full Danish population from 1999 to 2001, with an 11‐year follow‐up period, to examine the effects of hospital admission on subsequent psychiatric treatment trajectory, crime, and labor market outcomes for patients who seek treatment for the first time. We address differences between the counterfactual outcomes of individuals who are admitted and those who are not admitted by using an instrumental variable (IV): how the demand for treatment at a given hospital during the week leading up to an individual's first contact deviates from that hospital's maximum treatment demand. This measure, serving as a proxy for hospitals' actual occupancy rates, is unrelated to patient characteristics and strongly predicts whether an individual seeking treatment is admitted or not. Thereby, we identify the causal effect of an admission relative to no admission.

Our results show that in the short run, a psychiatric hospital admission reduces criminal behavior, particularly for those suffering from disorders such as psychosis or substance use‐related disorders. Thus, a failure to admit a marginal patient not only leaves the patient's immediate needs unaddressed—a concern voiced in The Lancet (2011)—but also results in negative externalities. We find that the short run crime reductions mainly arise from incapacitation of patients during admission. In the longer run, hospital admission continues to result in lower crime, but it also reduces patients' employment, both at the intensive and extensive margin, and their overall labor force participation rates, likely because psychiatric hospital admission is an eligibility criterion for receiving welfare benefits.

The study adds to the literature that estimates causal effects of psychiatric treatments on patients' later lives (Chang, Lichtenstein, D'Onofrio, Sjölander, & Larsson, 2014; Dalsgaard, Nielsen, & Simonsen, 2014) and to the studies finding that treatment of psychiatric illnesses is related to crime (e.g., Edwards, 2014). We also add to the literature studying incapacitation's crime reducing effect (e.g., Anderson, 2014; Appelbaum, 2001). To our knowledge, we are the first to study the effects of choosing to immediately admit and treat a patient the first time the patient contacts a psychiatric hospital. We show that the first hospital assessment affects subsequent treatment trajectories and may trigger persistent institutionalization. While some of the crime reduction from hospital admissions is temporary, even transient reductions to number of crimes have large economic and social returns for potential victims (Chalfin, 2016; McCollister, French, & Fang, 2010).

The paper progresses as follows: Section 2 introduces the institutional setting of psychiatric health care in Denmark. Section 3 describes the data and Section 4 describes the framework. Section 5 presents the results. Finally, Section 6 concludes.

## 2 PSYCHIATRIC HEALTH CARE IN DENMARK

In Denmark, health care is fully publicly funded and not directly affected by individual credit constraints. People may receive psychiatric treatment through two channels. The person can contact an emergency room at a hospital or contact their general practitioner (GP) who may refer the person for treatment.^2^ If the GP assesses that the person should receive psychiatric treatment, there are three options: (i) referral to a psychiatric practitioner (mildest cases), (ii) referral to a psychiatric hospital (mild to severe cases), (iii) or involuntary admission to a psychiatric hospital (most severe cases). In 2000, one‐third of psychiatric patients were in group (i), while groups (ii and iii) included the remaining two‐thirds (Bengtsson, 2011).

Psychiatric treatment consists of inpatient and outpatient treatments. Once a patient contacts a psychiatric hospital, his/her need for treatment is assessed by a psychiatrist who has three options: admit the patient to the hospital for inpatient services, suggest that the patient contacts outpatient services at his/her own initiative, or suggest that the patient contacts the GP again. Inpatient treatment takes place at hospitals or at designated facilities; outpatient treatment is delivered in the patient's local community.^3^ We analyze how hospital admission affects later use of these alternatives in Section 5.

In terms of urgency, intensity, and persistence of the treatment, there are large differences between inpatient and outpatient treatment modalities. On average, outpatient treatment during the period we study involved only one monthly contact with social services at a community health center and even less frequent contacts with physicians (see Bertelsen et al., 2008, p. 764; Petersen et al., 2005, p. 2 for details). Contact with outpatient services relied solely on the patient's own effort and initiative. Outpatient treatment was discontinued if patients failed to show up at scheduled appointments or respond to letters. Thus, the efficacy of outpatient treatment was closer to not receiving any treatment at all than it was to receiving inpatient treatment, and true *psychiatric* treatment could be considered effectively terminated once the patient was discharged from the hospital.

Danish psychiatric facilities have seen gradual budget cuts and capacity reductions over the past decades, especially with respect to inpatient treatment (Bengtsson, 2011). However, the period we study (1999–2001) is stable without any major reforms or reductions of adult psychiatric treatment possibilities, and no large policy‐driven national or regional supply shocks to inpatient treatment capacity occurred during that time. Appendix A provides a brief overview of the trends in number of inpatient beds showing a stable level of around 7.5–8 beds per 10,000 inhabitants in 1996–2001, followed by a reduction of around 33% from 2001 to 2011.

## 3 DATA

This section describes the data and main variables. We use the Danish national psychiatric register, which contains information on all contacts with Danish psychiatric facilities from 1980 to 2011—the date of initial contact, treatment (if any), location, hospital and ward of contact, the date and type of admission (if any), and diagnosis. We identify the first contact with the psychiatric care system and the relevant treatment information. Each observation contains a unique individual identifier (and a unique case number) allowing us to link information from the demographic, educational, labor market, and crime registers. We focus on individuals with their first contact between the age of 18 and 45 from 1999 to 2001; we have excluded pediatric and geriatric psychiatric treatments due to their often unique and specialized nature. Although age 45 is not typically considered geriatric, several psychiatric conditions that predominantly target the elderly have a nonnegligible risk of early onset. Also, those aged 46+ who do not suffer from the onset of geriatric cognitive disorders either have a long history of admissions (i.e., it is not their first contact) or generally have a very low labor market attachment with 50%–60% being on permanent disability pension at the time of their first contact. We also exclude those admitted through the criminal justice system, and those diagnosed with mental retardation, dementia, disorders of early psychological development, eating disorders, and nonorganic sexual dysfunctions in order to obtain a more homogeneous sample. Finally, we exclude the few countryside treatment facilities that treat fewer than 50 individuals per year. This results in a final sample of 24,277 individuals.

### 3.1 Outcome variables

We measure outcome variables for up to 11 years following first contact with a psychiatric facility. We measure crime by combining the exact date of first contact with a psychiatric hospital with the exact date of crime. We measure crime as both the accumulated number of convictions from first contact through the following 11 years and as the occurrence of convictions within specific time intervals. Using these dual crime measures allows us to assess both timing of effects from admission and total accumulated effect.

We define labor market outcomes, measured from 1 to 11 years after first contact, through three mutually exclusive categories: *employment*, *unemployment*, and *not in the labor force* (i.e., receiving welfare benefits and not in the labor market). Finally, we use the psychiatric register to identify the likelihood of subsequently contacting and being admitted to a psychiatric hospital. The tables presenting estimation results in the main text will also show the mean values of the outcomes in question.

### 3.2 Treatment variable and sample descriptions

Our explanatory variable of interest is a dummy variable equal to 1 if an individual is admitted as inpatient at a psychiatric care facility and 0 otherwise (comprising all outcomes where the individual is not immediately admitted to the facility). Thus, our treatment variable represents the foremost admission decision. Table 1 shows summary characteristics by admission status. 7349 out of the 24,277 individuals are admitted. The 30% who experience immediate admission stay, on average, 39 days at the hospital. The table also shows substantial differences on many observable characteristics across admission status. Columns 2–3 show that more women than men contact psychiatric hospitals, but men are more often admitted to psychiatric hospitals at first contact. Admitted patients are older, have committed more crime, have higher unemployment rates, and are less likely to be outside the labor force prior to first contact. Admitted patients have parents with less education than individuals who are not admitted, and individuals suffering from disorders associated with substance use, psychosis, schizophrenia, or adult‐onset affective disorders are more likely to be admitted. When comparing column 1 to column 4, we see that individuals who seek access to psychiatric treatment come from more disadvantaged backgrounds in numerous dimensions relative to the average population sampled with a similar age profile.^4^

**TABLE 1:** Descriptive statistics for sample

	(1)	(2)	(3)	(4)
	Full sample	Not admitted	Admitted	Representative sample
Admitted	0.303 (0.459)	0.000	1.000	‐
Days of admission	11.88 (106.813)	0.000	39.105 (191.379)	‐
Male	0.463 (0.499)	0.423 (0.494)	0.555^***^ (0.497)	0.509 (0.500)
Immigrant	0.126 (0.331)	0.126 (0.332)	0.124 (0.330)	0.081 (0.273)
Age	31.141 (7.535)	30.821 (7.522)	31.879^***^ (7.515)	31.995 (7.794)
Gross income in Year 1 (1000 DKK)	194.427 (130.355)	193.502 (131.852)	196.557^+^ (126.823)	264.456 (207.097)
Committed crime any time before this year	0.280 (0.449)	0.255 (0.436)	0.337^***^ (0.473)	0.138 (0.345)
Unemployment degree in Year 1	0.183 (0.305)	0.179 (0.304)	0.193^***^ (0.308)	0.071 (0.199)
Welfare dependency in Year 1	0.334 (0.383)	0.344 (0.387)	0.311^***^ (0.371)	0.215 (0.347)
Mother's months of schooling	125.239 (34.248)	126.928 (34.427)	121.348^***^ (33.510)	125.526 (38.394)
Father's months of schooling	135.892 (35.023)	137.235 (34.924)	132.801^***^ (35.058)	137.650 (40.440)
Mother's age at birth	26.206 (5.035)	26.215 (5.007)	26.188 (5.099)	25.360 (5.164)
Admitted in own municipality	0.164 (0.370)	0.166 (0.372)	0.157^+^ (0.364)	‐
Admitted in Copenhagen	0.193 (0.395)	0.207 (0.405)	0.161^***^ (0.368)	‐
Admitted in metropolitan area	0.152 (0.359)	0.150 (0.357)	0.158 (0.365)	‐
Disorder associated with substance use	0.152 (0.359)	0.125 (0.331)	0.213^***^ (0.410)	‐
Psychosis, schizophrenia	0.074 (0.262)	0.037 (0.188)	0.161^***^ (0.368)	‐
Affective disorder	0.199 (0.399)	0.180 (0.384)	0.243^***^ (0.429)	‐
Anxiety or stress‐related	0.444 (0.497)	0.504 (0.500)	0.305^***^ (0.460)	‐
Personality disorder	0.103 (0.305)	0.118 (0.322)	0.071^***^ (0.256)	‐
Affective/emotional, preadult origin	0.028 (0.164)	0.036 (0.187)	0.007^***^ (0.086)	‐
Observations	24,277	16,928	7349	716,411

*Notes*: Table shows the means and std. dev. of covariates for the full sample and divided by treatment status. *T*‐tests of differences between inpatient and outpatient subsamples. Far right column shows summary statistics of a randomly selected sample of individuals aged between 18 and 45 in 1999–2001. *Source*: Own calculations on data from Statistics Denmark. Standard deviation in parentheses.

^+^ *p* < 0.10; *p < 0.05; **p < 0.01; ***p < 0.001.

### 3.3 Instrumental variable

We use hospital‐specific contact intensity as an IV for psychiatric hospital admission. The IV is the relative deviation in the number of unique contacts at a given hospital in the 7 days prior to a treatment‐seeking individual's first contact relative to the maximum number of contacts at that hospital within any 7‐day period during the past year. The use of contact intensity as IV instead of occupancy rate is motivated by the fact that Danish public psychiatric hospitals almost always operate at or above official capacity to treat as many individuals as possible. The average occupancy rate for Danish psychiatric hospitals was 95.6% in 1999 (Sundhedsstyrelsen, 2001), which suggests constant admission probabilities close to zero. This is in stark contrast to the actual variation in admission probability, mainly because the underlying characteristics of admitted patients and how many that can be discharged to make room for new patients vary with past weeks' treatment demand. Also, admission on an ad hoc basis is always possible for a new patient in dire distress, even when hospitals formally operate at full capacity. Thus, actual observed contact intensity predicts more how likely a hospital is to admit an individual at a given day more precisely than the formal occupancy rate.

We use the psychiatric register to compute the IV. The numerator is the number of contacts within the week prior to each individual's first contact from 1999 to 2001 (excluding the day of an individual's contact so the individual does not affect the IV). Importantly, we only count individuals once within a given 7‐day period to avoid high admission thresholds affecting the number of contacts if the same patients keep reappearing. The numerator contains information on the total demand for psychiatric treatments at a given date at a given hospital. To control for hospital size and maximum treatment demand, we create a similar variable containing the maximum number of contacts within any 7‐day interval during the past year from the date of each individual contact. This is the denominator in our IV. For individual *i* contacting hospital *h* on day d=0, the IV equals:

(1) $$Z_{ih}=1-\frac{{\sum^{\text{​}}}_{d=-7}^{-1}{\text{Contacts}}_{\text{hd}}}{\text{max}\left({\sum^{\text{​}}}_{d=-7}^{-1}{\text{Contacts}}_{\text{hd}},\ldots ,{\sum^{\text{​}}}_{d=-365}^{-359}{\text{Contacts}}_{\text{hd}}\right)}$$

The instrument falls by construction between 0 and 1. A value of 0 indicates that the 7 days before an individual's first contact were the busiest week at that hospital during the last year (i.e., the hospital operates at high capacity). A value of 1 indicates that there were no contacts to the hospital during the past week (i.e., demand for beds was low, which given fixed supply increases likelihood of vacant beds). We obtain identification from variation between individuals and between hospitals. We do not take hospital fixed effects because it would lead us to compare patients at different treatment margins around each hospital's mean contact intensity, and not relative to the maximum levels, changing the interpretation of estimation results substantially.^5^ We cluster standard errors at the hospital level.

**FIGURE 1:**
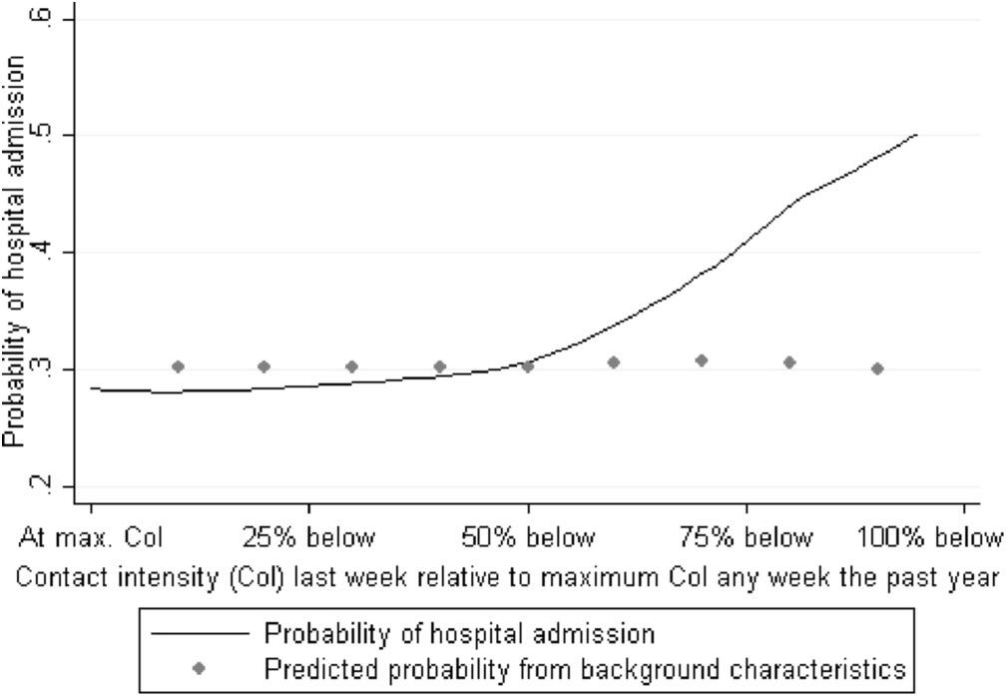
Probability of hospital admission across the instrument. The scatter plots show predicted probability of admission using covariates from Table 1 across the instrument using 0.1 bins. *Source*: Own calculations on data from Statistics Denmark

Figure 1 shows the probability of being admitted for different values of the IV. The figure also presents predicted probability of admission across the IV, estimated using the full set of covariates presented in Table 1. The probability of being admitted is unambiguously upward sloping across the entire interval of hospital‐specific contact intensity, while the predicted probability from observable characteristics is constant across different values of the IV.^6^ In the next subsection, we show formal balancing tests, and in Section 5, we present the first stage estimates.

### 3.4 Validity of instrument

Our key assumption is that the IV is unrelated to unobservable characteristics that affect outcomes, including (i) GPs' behavior, (ii) individuals' own characteristics, and (iii) general conditions such as business cycles and local labor market conditions. We test these potential confounding channels. If GPs postpone referrals or commence treatment themselves due to admission constraints, it would create an association between the IV and patient's number of visits to the GP prior to first contact. Panels A–D in Table SB.1 in the Appendix show estimates from regressions of GP and specialist physician visits on the IV. Because patients may postpone contacts to hospitals as a result of high contact intensity, we consider the number of visits during the year of initial contact as well as during the previous year. None of the estimates are significant on any conventional significance level; hence, prior to the first contact to a psychiatric hospital, the deviations in hospital contact intensity are not related to alternative treatment use.

We next test if the IV predicts whether a patient contacts the default hospital in his/her home county, also in Table SB.1 in the Appendix. This tests whether a first‐time patient tends to self‐select into a hospital with low contact intensity instead of contacting the hospital close to their home. We find no indications that patients self‐select into a particular hospital when the contact intensity there is low. We also perform the test using the difference in contact intensity between the actual contacted hospital and the hospital near where the patient lives. Again, we find no indications that patients self‐select into particular hospitals.

Another concern is endogeneity issues caused by systematic relationships between the IV and patient covariates, outcomes of interest such as crime and labor market outcomes, or local conditions. In Table SB.2 in the Appendix, we examine this (as motivated in Pei, Pischke, & Schwandt, 2019) by regressing the IV on our set of explanatory variables and information on previous crime and labor market attachment, indications on whether the patient's mother was ever admitted to account for intergenerational transmission and the average labor market conditions in the patients' area of residence to account for business cycles. Only 1 out of 24 balancing estimates is significant, so we consider it feasible that our key assumptions hold.

## 4 ECONOMETRIC FRAMEWORK

This study investigates the effects of admission to inpatient treatment at a psychiatric hospital versus nonadmission for an individual *i*—a person in psychiatric distress—on their subsequent outcomes. $D_{i}=\left\{0,1\right\}$ indicates nonadmission and admission, respectively, $X_{i}$ observable characteristics, and $Y_{i}$ the outcome of interest. Using a linear specification:

(2) $$Y_{i}=\alpha +X_{i}\text{'}\beta +\gamma D_{i}+\epsilon_{i}$$

where *γ* is our parameter of interest. It measures the effect of being admitted to a psychiatric hospital upon first contact relative to not being admitted. However, the error term $\epsilon_{i}$ is likely related to elements determining $D_{i}$, which would lead to biased estimates. To circumvent this, we employ an IV *Z*: the number of contacts the week prior to initial contact relative to the maximum number of contacts within a week during the past year. We use a two‐stage least squares (2SLS) approach with the following first stage equation:

(3) $$D=X_{i}\text{'}\pi_{1}+Z_{i}\text{'}\pi_{2}+u_{i}$$

A pivotal assumption is that the fraction of the number of weekly contacts relative to the maximum number of contacts within a given week during the past year is independent of unobservable characteristics affecting the outcome. While the assumption cannot be tested directly, the tests in Section 3.4 strongly support this. In addition to independence between the instrument and the unobservable characteristics, we also need a monotonic effect of the instrument on the endogenous regressor for all values of the instrument. Figure 1 illustrates that this is indeed the case (albeit we cannot confirm this with certainty because the monotonicity assumption applies at the individual level).

As *Z* likely constitutes an exogenous and monotonic instrument for *D*, we identify the average effect of being admitted for those whose treatment status changes from “no admission” to “admission” when *Z* increases to $Z^{\text{*}}$. To investigate potential heterogeneous treatment effects, we also estimate effects across characteristics such as gender, age, and criminal history (Table SB.6 in the Appendix).

We also report results from nonlinear two‐stage residual inclusion (2SRI) models for the analyses that examines crime, as these outcomes are somewhat rare, and nonlinear 2SRI models have been shown to perform well under such circumstances (Basu, Coe, & Chapman, 2018; Terza, 2017; Terza, Basu, & Rathouz, 2008). We estimate the 2SRI with a probit in the first stage, and a Poisson in the second stage for crime and subsequent contacts and admissions, and probit for the probability each of the three labor market outcomes. We calculate asymptotically correct standard errors following Terza (2017) and report the 2SRI estimates as average marginal effects. Furthermore, we estimate a two‐stage control function multinomial logit with the labor market outcomes as the discrete choices following Guevara (2015), where the first stage consists of an ordinary least squares (OLS) regression including the instrument on the right hand side. We then obtain the residuals from the first stage and include them in the second stage multinomial logit model (MNL) model as the control function. We obtain standard errors through bootstrapping.

## 5 RESULTS

### 5.1 First stage results

Table 2 presents the results of the first stage regression of our IV on the hospital admission dummy. Column 1 shows the first stage estimate without any control variables, column 2 shows the estimate with a set of socioeconomic and demographic control variables, and column 3 also includes controls for diagnosis. Column 4 shows the average marginal effect of the first stage as a probit model. Column 1 shows that going from highest contact intensity to the lowest increases the probability of admission by 22% points. The first stage coefficient is slightly lower after controlling socioeconomic, demographic, and diagnosis controls in columns 2 and 3, where going from the highest to the lowest contact intensity predicts a 17% points increase in admission probability. The small and insignificant differences in point estimates across the different specifications confirm the balancing of the covariates across the IV. The *F*‐value for the full specification in columns 3 and 4 exceeds the Staiger–Stock rule‐of‐thumb of 10 (Staiger & Stock, 1997), but just barely as the strength of the instrument is affected by the clustering of standard errors at the hospital level. In Table SB.3 in the Appendix, we report first stage estimates by subgroup showing a significant first stage for all groups, although not all *F*‐values are higher than 10. A result from a weak IV could lead to spurious conclusions as the first stage coefficient is the denominator in the second stage. To confirm that this is not driving our conclusions, Tables SB.4 and SB.5 in the Appendix show *reduced form* estimates where we regress the outcome on the instrument. The tables confirm that the 2SLS estimates arise from variation in admission to a psychiatric hospital from the instrument and not from a potentially weak IV.

**TABLE 2:** First stage estimation results

	OLS	OLS	OLS	Probit_AME_
Instrumental variable: Relative deviation between the number of contacts within the last week and the maximum number during a seven day period the last year	0.222^*^ (0.098)	0.168^**^ (0.059)	0.172^**^ (0.054)	0.171^**^ (0.051)
*F*‐value	5.11	8.12	10.19	11.89
Observations	24,277	24,277	24,277	24,277
SES and demographic controls	‐	X	X	X
Diagnosis controls	‐	‐	X	X

*Notes*: Table shows OLS regression and average marginal effect of Probit regression results of hospital admission (0/1) on the instrumental variable, hospital‐specific contact intensity, the week prior to the individual's initial contact. Standard errors clustered by hospital and month in parentheses. Socioeconomic and demographic controls include: gender (dummy), age at adm., mother's age at birth, mother's years of schooling, father's age at birth, father's years of schooling, mother has prior psych. history (dummy), admitted in own municipality (dummy), greater Copenhagen area (dummy), other metropolitan area (dummy), in regions Funen or Jutland (dummy), year dummies. Diagnosis controls include: Dummies for each F. diagnosis category from International Classification of Diseases, 10th version. *Source*: Own calculations on data from Statistics Denmark.

Abbreviations: AME, average marginal effects; OLS, ordinary least squares; SES, Socioeconomic.

^+^ *p* < 0.10; ^*^ *p* < 0.05; ^**^ *p* < 0.01; ^***^ *p* < 0.001.

### 5.2 Main results for crime

Table 3 shows OLS, 2SLS, and 2SRI estimates of the effect of an admission at first contact with a psychiatric hospital on subsequent crimes.^7^ Panel A reports effects of a hospital admission on the crime measured between the specific time intervals^8^ and inform us whether hospital admission changes subsequent behavior at a given point in time. Panel B shows results for accumulated number of crimes from first contact in order to estimate the degree to which an admission causes persistent change.

The OLS estimates show that hospital admission is positively associated with subsequent crime. When we focus on the 2SLS and 2SRI estimates, however, we see a significant and substantial decrease in crime. We see from Panel B that the effect accumulates and is at least borderline significant up until 11 years after first contact. The table further shows that the sign of the 2SLS and 2SRI estimates are similar but the latter estimates are more precise. Panel A in Table SB.6 in the Appendix shows the corresponding estimates by subgroup. Psychiatric hospital admission significantly reduces crime in the shorter run for males (column 1), with the effect driven by young individuals (column 3) and those who have previously committed crime (column 6). Table SB.7 in the Appendix reports results where we separate crime by type: *property crime*, *violent crime*, and the residual *other crime* (including drug‐related offenses). The overall crime reductions are driven by decreases in property crime and other crimes. Thus, the positive spillovers to public safety from hospital admissions do not come from fewer victims of violence, but rather from fewer crimes potentially associated with obtaining means to acquire substances to self‐medicate with.

In conclusion, our results show that a hospital admission leads to significant reductions in crime—both in the short and long run. Thus, hospital admission appears to reduce patients' adverse behaviors.

### 5.3 Mechanisms and dynamics

#### 5.3.1 Incapacitation

To examine whether incapacitation explains the effects of an admission, we reestimate the model with crime as outcome. Instead of setting time zero at date of first contact, we set time zero at date of discharge. Thus, for admitted patients, 12 months now refer to 12 months after discharge and not 12 months after initial contact. Table SB.8 in the Appendix shows the results. In columns 1–2, we report results where we define time 0 at the date of discharge, and in columns 3–4, we reprint as comparison the estimates from Table 3, where time 0 is the date of initial contact. Panel A of Table SB.8 shows that there is no significant reduction to crime when we measure crime from the day of the discharge instead of the day of contact/admission for neither 2SLS nor 2SRI estimates. The 2SLS estimates are insignificant and close to zero (although confidence levels are wide) with 2SRI estimates being larger in magnitude. The results suggest that incapacitation during hospital admission caused at least some of the crime reduction.

**TABLE 3:** Effect of admission on crimes

Time	Mean	OLS	2SLS	Poisson	AME_Poisson_	2SRI	AME_2SRI_
A: Within time intervals							
3 months	0.021	0.009^***^ (0.002)	−0.054^+^ (0.030)	0.352^**^ (0.109)	0.004	−3.623^*^ (1.705)	−0.039
6 months	0.021	0.006^**^ (0.002)	−0.036 (0.034)	0.256^***^ (0.060)	0.003	−2.564^+^ (1.378)	−0.029
1 year	0.041	0.002 (0.004)	−0.014 (0.054)	0.031 (0.083)	0.001	−2.072 (1.288)	−0.046
2 years	0.070	0.020^***^ (0.005)	−0.095 (0.074)	0.243^***^ (0.059)	0.010	−2.133^+^ (1.111)	−0.087
3 years	0.067	0.030^***^ (0.005)	0.059 (0.070)	0.388^***^ (0.058)	0.015	−0.062 (0.758)	−0.002
7 years	0.220	0.074^***^ (0.013)	−0.360 (0.254)	0.277^***^ (0.060)	0.036	−2.179^+^ (1.179)	−0.279
11 years	0.107	0.047^***^ (0.009)	−0.213 (0.151)	0.333^***^ (0.053)	0.022	−1.985^+^ (1.028)	−0.133
B: Accumulated from first contact							
3 months	0.021	0.009^***^ (0.002)	−0.054^+^ (0.030)	0.352^**^ (0.109)	0.004	−3.623^*^ (1.705)	−0.039
6 months	0.043	0.015^***^ (0.004)	−0.090^+^ (0.052)	0.304^***^ (0.067)	0.007	−3.093^*^ (1.288)	−0.068
1 year	0.084	0.017^**^ (0.006)	−0.105 (0.086)	0.171^***^ (0.065)	0.008	−2.607^*^ (1.154)	−0.117
2 years	0.154	0.037^***^ (0.008)	−0.199 (0.135)	0.203^***^ (0.065)	0.018	−2.385^*^ (1.035)	−0.205
3 years	0.221	0.068^***^ (0.011)	−0.140 (0.175)	0.260^***^ (0.047)	0.033	−1.665^+^ (0.861)	−0.209
7 years	0.441	0.138^***^ (0.020)	−0.584^+^ (0.345)	0.267^***^ (0.047)	0.068	−2.042 (0.878)	−0.517
11 years	0.589	0.181^***^ (0.024)	−0.713^+^ (0.418)	0.281^***^ (0.043)	0.090	−1.892^*^ (0.813)	−0.608
Observations		24,277	24,277	24,277	‐	24,277	‐
SES and demographic controls		X	X	X	‐	X	‐
Diagnosis controls		X	X	X	‐	X	‐

*Notes*: Table shows outcome means and OLS, 2SLS, and average marginal effects from 2SRI regression results of hospital admission (0/1) on subsequent crimes. 2SRI for crime estimated with Poisson for second stage. Time 0 is month of initial contact. Panel A shows results for between periods incidents (i.e., 0–3 months, 3–6 months, 6–12 months, 1–2 years, 2–3 years, 3–7 years, 7–11 years). Panel B shows results for incidences accumulated from time of first contact. S.E. clustered by hospital in parentheses. Socioeconomic and demographic controls include: Gender (dummy), age at adm., mother's age at birth, mother's years of schooling, father's age at birth, father's years of schooling, mother has prior psych. history (dummy), admitted in own municipality (dummy), greater Copenhagen area (dummy), other metropolitan area (dummy), in regions Funen or Jutland (dummy), year dummies. Diagnosis controls: Dummies for each F. diagnosis category from International Classification of Diseases, 10th version. *Source*: Own calculations on data from Statistics Denmark.

Abbreviations: AME, average marginal effects; OLS, ordinary least squares; 2SLS, two‐stage least squares.

^+^ *p* < 0.10; ^∗^ *p* < 0.05; ^∗∗∗^ *p* < 0.001.

#### 5.3.2 Substitution between treatment alternatives?

Next, we examine how admission upon first contact shapes the later treatment trajectory. It is difficult to judge whether subsequent contact behavior should be viewed positively or negatively as, for example, a reduction in later contacts and admissions could reflect the patient being restored to health or inadequate access to treatment. It is, nevertheless, important to explore the dynamic consequences of admission on later health care use to better understand how the initial admission decision affects later health care expenses and mediate effects on other patient outcomes.

Panels A and B in Table 4 show the estimated effects of a hospital admission on the probability of contacting or being admitted to a psychiatric hospital again. We present outcomes measured the week immediately after first contact to investigate the short run response to (not) being admitted, and then in 3‐month intervals from month 1–3, 4–6, and 10–12 after first contact with a psychiatric hospital, with the outcomes measured as dummies equal to 1 if contact/admission took place in a given 3‐month period. An admission is significantly associated with subsequently contacting and being admitted to a psychiatric hospital again, but the 2SLS estimates show that in the very short run, a hospital admission reduces the likelihood that the patient contacts a hospital again the following week by 20% points. Thus, individuals who seek help but are not admitted are 20% points more likely to reach out for help again within a short period of time. However, the table also shows that this effect is not driven by contacts with another hospital and, as evidenced from the first row in Panel B, those who are not admitted at first contact are not more likely to be admitted anyway shortly after.

**TABLE 4:** Effect of admission on subsequent contact and admission to psychiatric hospitals and use of GP and specialist practitioner

Time	Mean	OLS	2SLS
A: New contact			
1 week	0.099	0.040^*^ (0.019)	−0.200^+^ (0.110)
1 week, contact to new hospital	0.016	0.014^**^ (0.005)	0.030 (0.045)
3 months	0.301	0.253^***^ (0.007)	0.216 (0.187)
6 months	0.370	0.087^***^ (0.005)	0.041 (0.124)
12 months	0.421	0.053^***^ (0.004)	0.000 (0.051)
B: New admission			
1 week	0.032	0.017^*^ (0.006)	0.042 (0.057)
3 months	0.128	0.118^***^ (0.005)	0.212^*^ (0.104)
6 months	0.172	0.055^***^ (0.004)	0.025 (0.079)
12 months	0.211	0.045^***^ (0.003)	0.026 (0.037)
C: Use of GP or specialist practitioner			
GPs, Year 1	7.556	0.022 (0.069)	−2.475 (2.086)
Specialists, Year 1	0.513	−0.037^*^ (0.019)	−0.649^+^ (0.348)
GPs, Year 2	7.076	0.002 (0.066)	−1.342 (1.413)
Specialists, Year 2	0.356	−0.031^*^ (0.014)	−0.241 (0.201)
Observations	24,277	24,277	24,277
SES and demographic controls	‐	X	X
Diagnosis controls	‐	X	X

*Notes*: Table shows outcome means and OLS, and 2SLS regression results of hospital admission (0/1) on probability of subsequently contacting a psychiatric hospital again, the probability of subsequently being admitted to a psychiatric hospital again, and the number of contacts/treatments at GPs or specialist practitioner. Time 0 is month of initial contact. Table shows results for quarterly contact and admission rates, that is, from month 0–3, 3–6, 9–12, and for GPs and specialists, the first 2 years following first contact to a psychiatric hospital. Standard errors clustered by hospital in parentheses. Socioeconomic and demographic controls include: Gender (dummy), age at adm., mother's age at birth, mother's years of schooling, father's age at birth, father's years of schooling, mother has prior psych. history (dummy), admitted in own municipality (dummy), greater Copenhagen area (dummy), in regions Funen or Jutland (dummy), other metropolitan area (dummy), year dummies. Diagnosis controls include: Dummies for each F. diagnosis category from International Classification of Diseases, 10th version. *Source*: Own calculations on data from Statistics Denmark.

Abbreviations: GP, general practitioner; OLS, ordinary least squares, SES, Socioeconomic; SLS, stage least squares.

^+^ *p* < 0.10; ^∗^ *p* < 0.05; ^∗∗^ *p* < 0.01; ^∗∗∗^ *p* < 0.001.

The estimates also show that an admission increases the probability of later being admitted to a psychiatric hospital by 21% points within the first 3 months from first contact. These results are in stark contrast with the immediate response to admission in the week after first contact and suggest some degree of institutionalization where doctors may be more prone to readmit a former patient whom they have recently treated. Yet, beyond 3 months after the initial contact there are no significant effects of admission at first contact on later admission probability.

We thus find no evidence that admission upon first contact with a psychiatric hospital results in long run improvements as measured by later treatment patterns. Rather contrary, we find a significant short run spike in the likelihood of readmission during the first 3 months after first contact.

Panel C presents estimated effects of psychiatric hospital admission on subsequent treatments received from GPs and specialist practitioners in the first 2 years after first contact with a psychiatric hospital. All 2SLS estimates are negative thereby elucidating the counterfactual to hospital admission. A priori rejected individuals could receive no treatment, outpatient treatment, or treatment from GPs or specialist practitioners. The results in the first row of Panels A and B in combination with Panel C clearly suggest that treatment by GPs and specialist practitioners was the most predominant substitute used. However, the further results in Panels A and B also suggest that no hospital admission versus hospital admission triggers a more fundamental substitution between one treatment alternative for another, as admitted individuals are more likely to experience subsequent hospital admissions.

The substitute that patients use may have longer run consequences on other margins beyond choice of subsequent medical treatment. Patients may also increase use of welfare services because admitted patients are increasingly unavailable for the labor market for a longer period, and thus are in need of income support. We investigate this relationship next.

### 5.4 Hospital admission, employment, and welfare dependency

Admission to a psychiatric hospital is an eligibility criterion for disability pension and early retirement. Hospital admission may, therefore, reduce a patient's labor market attachment. Table 5 reports estimates for the effect of admitting a patient to a psychiatric hospital at first contact on the probability of subsequent employment, unemployment, and being out of the labor force the following 11 years using multinomial logit regression of the labor market outcomes regressed on a control function (2SCF) obtained from at first stage following Guevara (2015). We also report results from a MNL without adjusting for endogeneity.

**TABLE 5:** Effect of admission on labor market outcomes

	Time	Mean	MNL	AME_MNL_	2SCF	AME_2SCF_
Reference: Employment	1 year	0.521	Ref.	−0.054	Ref.	−0.068
	2 years	0.509	Ref.	−0.049	Ref.	−0.102
	3 years	0.501	Ref.	−0.053	Ref.	−0.195
	7 years	0.535	Ref.	−0.050	Ref.	−0.206
	11 years	0.462	Ref.	−0.061	Ref.	−0.168
A: Unemployment	1 year	0.179	0.214^***^ (0.059)	0.016	0.105 (0.516)	−0.009
	2 years	0.173	0.189^***^ (0.056)	0.013	0.143 (0.538)	−0.016
	3 years	0.166	0.179^***^ (0.043)	0.009	0.805 (0.542)	0.055
	7 years	0.097	0.071 (0.56)	−0.004	1.169^+^ (0.668)	0.078
	11 years	0.127	0.136^***^ (0.038)	−0.001	0.345 (0.612)	−0.010
B: Not in labor force	1 year	0.300	0.246^***^ (0.048)	0.038	0.397 (0.432)	0.077
	2 years	0.318	0.222^***^ (0.053)	0.035	0.591 (0.435)	0.119
	3 years	0.333	0.253^***^ (0.056)	0.043	0.881^*^ (0.438)	0.140
	7 years	0.367	0.259^***^ (0.047)	0.053	0.827^*^ (0.402)	0.128
	11 years	0.411	0.316^***^ (0.045)	0.063	0.874^*^ (0.428)	0.178
Observations		‐	24,277	‐	24,277	‐
SES and demographic controls		‐	X	‐	X	‐
Diagnosis controls		‐	X	‐	X	‐

*Notes*: Table shows outcome means, coefficients and average marginal effects from a multinomial logit (MNL) and a multinomial logistic two‐stage control function (2SCF) regression for the effect of hospital admission (0/1) on probability of being in employment, being actively unemployed, or not being in the labor force. Time 0 is month of initial contact. 2SCF standard errors obtained from 1000 bootstrap replications. Socioeconomic and demographic controls include: Gender (dummy), age at adm., mother's age at birth, mother's years of schooling, father's age at birth, father's years of schooling, mother has prior psych. history (dummy), admitted in own municipality (dummy), greater Copenhagen area (dummy), other metropolitan area (dummy), in regions Funen or Jutland (dummy), year dummies. Diagnosis controls include: Dummies for each F. diagnosis category from International Classification of Diseases, 10th version. *Source*: Own calculations on data from Statistics Denmark.

Abbreviations: AME, average marginal effects; MNL, multinomial logit model; 2SCF, two‐stage control function; SES, Socioeconomic.

^+^ *p* < 0.10; ^∗^ *p* < 0.05; ^∗∗^ *p* < 0.01; ^∗∗∗^ *p* < 0.001.

The estimates show that admission leads to a reduction in employment and an increase in labor force exits during the following years. For example, 3 years after first contact, an admission leads to a 20% points reduction to employment, and a 14% points increase to the likelihood of not being in the labor force. These effects persist up to 11 years after first contact. Although the estimates for labor market outcomes appear large at first, they should be viewed in the light of eligibility criteria for welfare benefit reception. Social services use a psychiatric hospital record as a strong indicator of a person's eligibility to receive benefits without labor market requirements. Yet, Table 5 does not clarify how we should think of the negative relationship between hospital admission relative to the counterfactual state with no hospital admission. Are individuals at a constant or positive trajectory, which breaks downward following a hospital admission, or are the compliers already on a downward trend with deteriorating labor market attachment, which is then amplified by an admission?

To further elucidate this, Figure SB.2 in the Appendix shows the potential outcomes for compliers' labor market outcomes. We estimate this following Abadie (2003) and describe the estimation procedure in detail in Appendix A. The figure shows the labor market outcomes for the individuals for whom the instrument determined whether they were admitted or not (i.e., the compliers) if they were admitted, $Y_{1}$, and if they were not admitted, $Y_{0}$. From the figure, we see that employment rates fall for the first 2 years following the first contact with a psychiatric hospital, disregarding whether the patient is admitted or not (Figure SB.2a). In the longer run, however, the employment rate continues to decrease to less than 40% for those who were admitted, while it is stable around 60% for those who were not admitted. Furthermore, unemployment rates remain relatively stable for both groups (Figure SB.2b), while admitted patients experience a consistently deteriorating labor market attachment with around 50% being out of the labor force 11 years after the first contact (Figure SB.2c).

At first glance, the results for labor market outcomes suggest that the uptake in welfare claims is almost matched 1:1 by lower employment, but Figure SB.2 paints a more complex picture of several mechanisms at play. Psychiatric patients experience a deteriorating trajectory irrespective of hospital admission, but the admission amplifies this trajectory likely via welfare eligibility rules.

Moreover, hospital admission may not only affect employment at the extensive margin but also at the intensive margin. To assess this potential adjustment, we define three categories of annual labor earnings: $0 (corresponding to not being employed), $1000–$30,000 (suggesting part‐time work), and >$30,000 (suggesting full‐time work). Table SB.9 in the Appendix presents the effects of hospital admission on the probability of falling into each of the three categories during the 3 years after initial contact. The table shows that hospital admission makes full time work less likely. While the table illustrates an increase in nonemployment (corresponding to Table 5) following hospital admission, a sizeable fraction also moves from earning levels corresponding to full‐time work to labor earning levels suggesting part‐time work. For example, hospital admission makes it 20% points less likely that individuals earn above $30,000 on annual basis 2 years after the first contact and 11% points more likely that they instead earn between $1000–$30,000.

### 5.5 Heterogeneity by diagnosis

A further source of heterogeneity is the mental health problems that patients struggle with some often result in externalizing behavior, while others mainly result in internalizing behavior. To explore this potential heterogeneity, Table 6 shows the estimated effects on all main outcomes studied diagnosis category. On the one hand, the table shows that admission affects crime for patients suffering from problems related to psychosis, schizophrenia, substance use, mania, and personality and affective disorders. There are no significant effects on crime for patients suffering from anxiety or stress. On the other hand, the latter patient group is driving the effects of subsequent contact and admission to a hospital and employment. Here, admission to a psychiatric hospital at the first contact reduces the probability of subsequently being in employment by almost 30% points. Admission appears to lower adverse behaviors for patients diagnosed with more externalized afflictions, whereas it increases hospitalizations for patients with more internalized afflictions.

**TABLE 6:** Immediate and long run effects of admission, by diagnosis at first contact

		Anxiety or stress‐related			Psychosis, schizophrenia substance use‐related, mania, personality disorder affective disorder		
	Time	OLS	2SLS	AME_2SRI_	OLS	2SLS	AME_2SRI_
A: Crimes	3 months	0.010^**^ (0.003)	−0.002 (0.031)	−0.005 (0.008)	0.008^*^ (0.003)	−0.092^+^ (0.053)	−0.067^+^ (0.035)
	7 years	0.144^***^ (0.024)	0.025 (0.259)	−0.040 (0.131)	0.136^***^ (0.029)	−1.051^+^ (0.537)	−0.875^*^ (0.441)
	11 years	0.183^***^ (0.029)	−0.026 (0.337)	−0.069 (0.164)	0.180^***^ (0.035)	−1.223^+^ (0.635)	−0.999^*^ (0.501)
B: New contact	3 months	0.329^***^ (0.020)	0.308 (0.365)	0.180 (0.265)	0.390^***^ (0.017)	−0.452 (0.496)	−0.329 (0.441)
	3 years	0.774^***^ (0.055)	0.029 (0.944)	−0.232 (0.711)	1.306^***^ (0.065)	0.271 (1.269)	−0.355 (1.141)
C: New admission	3 months	0.134^***^ (0.010)	0.372^*^ (0.148)	0.234^**^ (0.073)	0.179^***^ (0.010)	0.162 (0.235)	0.140 (0.178)
	3 years	0.405^***^ (0.028)	0.543 (0.340)	0.309^+^ (0.187)	0.818^***^ (0.040)	0.816 (0.663)	0.364 (0.543)
D: Employment	1 year	−0.060^***^ (0.012)	−0.235^+^ (0.142)	−0.188 (0.141)	−0.052^***^ (0.009)	0.086 (0.176)	0.162 (0.173)
	7 years	−0.048^***^ (0.011)	−0.385^*^ (0.163)	−0.323^*^ (0.144)	−0.053^***^ (0.009)	−0.048 (0.142)	0.009 (0.152)
	11 years	−0.083^***^ (0.012)	−0.282^*^ (0.116)	−0.302^*^ (0.135)	−0.050^***^ (0.009)	−0.061 (0.119)	0.023 (0.115)
Observations		10,775	10,775	10,775	13,502	13,502	13,502
SES and demographic controls		X	X	X	X	X	X
Diagnosis controls		‐	‐	‐	X	X	X

*Notes*: Table shows OLS, 2SLS, and average marginal effects of 2SRI regression results of hospital admission (0/1) on crime convictions, repeat contact and admission to psychiatric hospital, probability of being in employment (vs.) being unemployed or not being in the labor force). Time 0 is month of initial contact. Standard errors clustered by hospital in parentheses. Socioeconomic and demographic controls include: Gender (dummy), age at adm., mother's age at birth, mother's years of schooling, father's age at birth, father's years of schooling, mother has prior psych. history (dummy), admitted in own municipality (dummy), greater Copenhagen area (dummy), other metropolitan area (dummy), in regions Funen or Jutland (dummy), year dummies. Diagnosis controls include: Dummies for each F. diagnosis category from International Classification of Diseases, 10th version. *Source*: Own calculations on data from Statistics Denmark.

Abbrevaitions: AME, average marginal effects; OLS, ordinary least squares; SES, Socioeconomic.

^+^ *p* < 0.10; ^∗^ *p* < 0.05; ^∗∗^ *p* < 0.01; ^∗∗∗^ *p* < 0.001.

## 6 CONCLUSION

In this study, we use hospital‐specific contact intensity during the weeks prior to a patient's first contact with the mental health system to identify the causal effects of admitting a patient to a psychiatric hospital at first contact compared to not admitting them at first contact. Using a sample of all first contacts to psychiatric hospitals by individuals aged 18–45 between 1999 and 2001 in Denmark, we find that admitting a first‐time patient has large but ambiguous effects.

A psychiatric hospital admission lowers criminal behavior in the first 3–6 months after the admission. These reductions are driven by males, individuals with a criminal background, and those with a diagnosis such as psychosis or schizophrenia, and likely emerge partly as a result of incapacitation. Furthermore, an initial hospital admission likely demarcates trajectories in the type of welfare dependency and use. In the longer run, the initial hospital admission continues to result in lower crime. It also increases the likelihood of experiencing a subsequent admission, and those who are not admitted initially appear to substitute other treatment alternatives such as treatments by specialist practitioners. Furthermore, admission increases welfare reception and lowers employment, likely because a psychiatric hospital admission is an eligibility criterion for welfare benefits.

Mental health problems are causes of strain and distress, and they inflict large costs to patients and society in general. To reduce the adverse impact of mental disorders, society should aim at optimizing the treatment possibilities for patients. Our study not only shows that this is not a simple task, but it also emphasizes the likely consequences of failing to do so. Not only are patients affected, but so are potential victims of crimes and public finances.

## CONFLICT OF INTEREST

The authors declare that they have no conflict of interest.

## Supporting information

Supplemetary Material

## ENDNOTES

^2^
GPs may also prescribe most antidepressants, but only do so in mild cases (WHO, 2011b). The Danish prescription practice stands in contrast to practices in, for example, the United States where GPs may prescribe a broader range of psychopharmaceuticals (Mojtabai & Olfson, 2011).

^3^
Outpatient treatment, where individuals are treated while remaining at home, is coordinated by local social services together with psychiatrists and governed by the individual municipalities. Hoff et al. (2012) provide an overview of the Danish system compared to the English and the Dutch. Except for the funding schemes, the two strands of treatment possibilities resemble those of many OECD countries.

^4^
Comparison sample also obtained from the registry data.

^5^
Consider two hospitals. Hospital A's contact intensity is on average high, and hospital B's is on average low (with a few outlier‐weeks with many contacts). Hence, Hospital A will likely be constrained in the room for new admissions in many weeks, while Hospital B will not. In weeks where Hospital A is above average (i.e., close to the maximum), there will be no room, whereas variation around the low mean in Hospital B will not matter for admission probability. Yet, taking hospital fixed effect would cause us to align the different hospital‐specific margins as though they were the same, which could compromise the validity and monotonicity of the instrument.

^6^
While the figure suggests a nonlinear relationship between the IV and admission probability, any such nonlinearity is not sufficiently strong to increase the explanatory power of the IV when we include higher order polynomials in the first stage because the seemingly nonlinear relationship is located where the data support is weakest. This is illustrated by a comparison of admission probability across the IV in Figure 1 and the distribution of the IV in Figure SB.1.

^7^
We have estimated all the effects of admission on all outcomes while including regional fixed effects to control for any differential trends in the demand for care or the resources available for psychiatric care. Our findings are robust to this inclusion, as the estimates do not change qualitatively or significantly.

^8^
1–3 months, 3–6 months, 6 months–1 year, 1–2 years, 2–3 years, 3–7 years, 7–11 years from first contact.
