# Supplementary material for: Psychiatric hospital admission and later crime, mental health, and labor market outcomes
Source: Health Econ. 2020 Nov 2;30(1):165–79. doi: 10.1002/hec.4186 (PMC7756892; doi:10.1002/hec.4186)
Supplement: Supplementary file 1 — Supplemetary Material [file HEC-30-165-s001.pdf]

A Data Appendix

First, this section provides a brief overview of the trends in the number of patients and inpatient capacity at psychiatric hospitals during the period studied. Second, the section describes the data construction in detail, as a supplement to section 3 from the main text. And finally, we detail how we estimate the counterfactual outcomes of compliers in section 5.4.

Trends in Psychiatric Hospital Treatments

Figure A.1 shows the number of inpatient beds in Danish psychiatric hospitals per 1,000 inhabitants and the number of 20 to 64 year old patients per 1,000 persons in that age group between 1996 and 2011.

The Danish psychiatric system gradually downsized the number of inpatient beds from 1996–2011. The reductions from 1997 to 2001 were relatively small, from 0.80 in 1997 to 0.75 in 2001. From 2002 and onwards, the reductions increased in magnitude. In 2011 there were 0.53 beds per 1,000 inhabitants. The figure also shows that the number of adult inpatients between the age of 20 and 64 increased from 2.36 in 1996 to 3.05 in 2004, only to stagnate at around 2.90 until 2011. The downward trend in the number of psychiatric beds resembles that of many Western countries (WHO, 2011). The reductions to inpatient capacities relative to the number of treated patients moved treatment from a hospital setting to the patients' home environments while also reducing the costs of treatment.

Construction of Mental Health Data

We constructed the mental health data using the Danish national psychiatric register (a subset of the Danish patient register (LPR)) made available by Statistics Denmark. We study the period 1999 to 2001, and only consider patients who had their first contact in this

Figure A.1: Psychiatric beds and admissions, 1996-2011

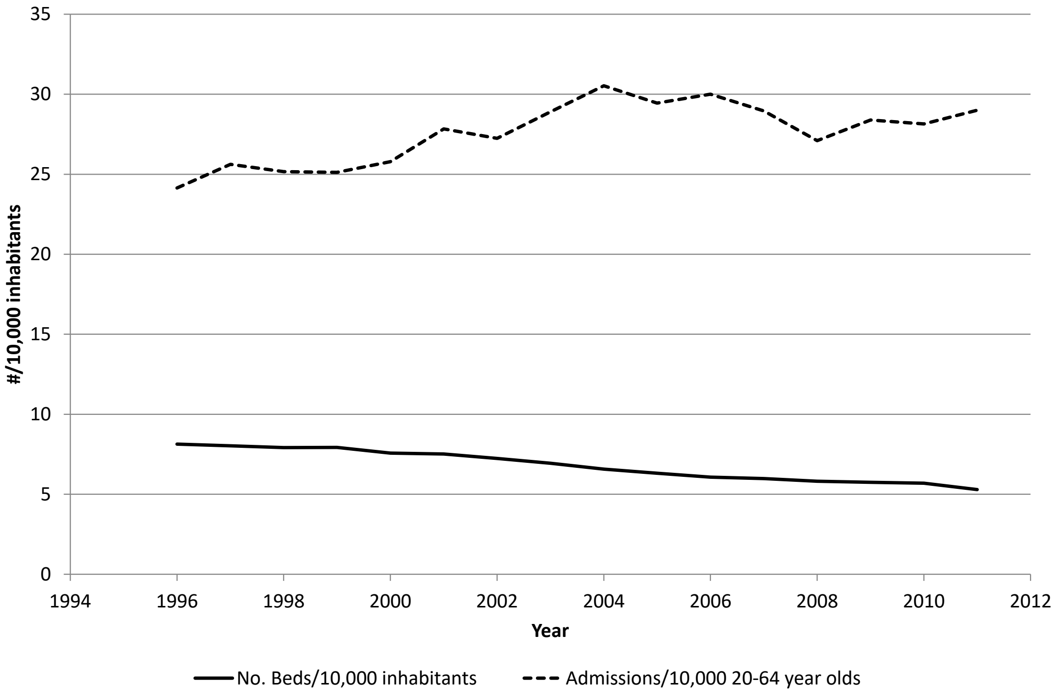

Source: Own calculations on data from Statistics Denmark.

period. This yields 50,439 patients. Next, we only include individuals who had their first contact between the ages of 18 and 45 as we do not wish to focus on child or geriatric psychiatric treatment as admission or non-admission of these patients is not likely to influence the outcomes we are interested in. Also, once in contact with a psychiatric care facility, different conditions apply for these two groups relative to the average adult population. The age limitation reduces the sample from 50,439 to 31,248. We discard individuals who are diagnosed with mental retardation, dementia, or disorders of early psychological development because these patient groups suffer from chronic disorders and have little or no labor market attachment. We also discard individuals who are diagnosed with eating disorders or non-organic sexual dysfunctions in order to obtain a more homogeneous sample. Individuals who enter treatment via the criminal justice system are also discarded. Finally, a few coun-

tryside treatment facilities only treat 10-50 individuals per year and we discard individuals who contact these facilities in order to not skew our instrument. Limiting the diagnoses used, disregarding criminal justice court ordered psychiatric treatments, and setting a lower threshold for facility size leave us with a final sample of 24,277 individuals. The final sample contains the following ICD-10 diagnosis categories:

(a) F10-F19: Mental and behavioral disorders due to psychoactive substance use

(b) F20-F29: Schizophrenia, schizotypal and delusional disorders

(c) F30-F39: Mood (affective) disorders

(d) F40-F48: Neurotic, stress-related and somatoform disorders

(e) F60-F69: Disorders of adult personality and behavior

(f) F90-F98: Behavioral and emotional disorders with onset usually occurring in childhood and adolescence

(g) F99: Unspecified mental disorder

We control for each diagnosis category. Only 52 people in the data receives a diagnosis in the range F90-F98, so they are grouped together with F99 diagnoses. Results do not change if we group them apart.

We define the outcome of a contact on the basis of a patient's experiences across an entire day. I.e., if a patient contacts a psychiatric emergency ward and later in the day is admitted to a normal psychiatric ward, that is treated as one incident (contact) leading to an admission. In order not to exclude people who show up late in the day, we use the same procedure for patient who have their first contact at an emergency ward at day one and are admitted to a normal word at day two. We distinguish between admission to inpatient care, admission into outpatient care, and no admission. We define the treatment variable as admission to inpatient care.

Construction of the Instrument

We used the Danish National Psychiatric Register to obtain the number of unique individual contacts to each hospital each day for the period 1998-2001 for all ages and types of diagnoses. We included the year 1998 in order to construct the instrument as a contact intensity measure as shown in Equation 1. We then computed the number of weekly contacts separately for the two seven day periods prior to a person's first contact by aggregating the number across day -1 to -7, and day -8 to -14. This gave us our numerators. In order to obtain the denominator, which measures the seven day period with the highest number of contacts within the last 365 days, we calculated the aggregated number for all successive seven days combination from the day prior to an individual's contact and going backward 365 days. We then used the highest number of contacts measured across a seven day period as the denominator. The two fractions made up the instruments.

Construction of Covariates

We constructed covariate data using a number of databases. Unique individual identification numbers allowed us to directly link observations across registers, and also link information on patients with information on their parents and potential spouse. We used the following databases:

(a) Danish demographic database (FAIN), from where we obtained age, ethnicity, and municipality of residence for year of first contact.

(b) The register based labor force database (RAS), unemployment register (CRAM), and the Danish Rational Economic Agents Model (DREAM) to obtain information on unemployment degree and public dependency degree the year prior to first contact.

(c) The database of individual usage of health services (SYIN) to obtain information on admissions to hospital for somatic reasons as well as visits to GP and specialist doctors

the year prior to first contact.

(d) The educational database (UDDA) to obtain information on parental schooling. We obtain the information for each year since 1981, and use the information recorded closest to the year of first contact.

(e) The criminal justice databases on convictions (KRAF) and indictments (KRSI) to obtain information on prior criminality. We use information of whether an individual ever has received a conviction for a non-traffic related offense or felony from 1980 and until the year before first contact.

(f) The income database (INDK) to obtain information on gross income reported the year before first contact.

Construction of Outcomes

We measured all outcomes for the following three years after first contact an in three month windows from month 1-3, 4-6, 10-12, 22-24, 34-36 from first contact. The following paragraphs provide details on how we constructed the different measures.

Crime Using the criminal justice databases on convictions (KRAF) and indictments (KRIN) we constructed the crime variable as a quarterly count variable that aggregated all criminal convictions received for crimes committed within three month windows (as detailed above) and accumulated from first contact and the following 36 months. We excluded traffic violations.

Contacts and Admissions We followed each patient in the Danish national psychiatric register data for the 36 subsequent months after first contact. Contact was constructed as a dummy, indicating if the patient had new subsequent contacts to a psychiatric hospital within three month windows (as detailed above). Admission was constructed as a subset

of the contact dummy, indicating if the patient was admitted as an inpatient following a contact during the periods in question.

Labor Market Outcomes We use the register based labor force database (RAS) to obtain annual information on individual's labor market position for the three years after contact. We define three categories: employed, unemployed, and outside the labor force. Employed entails any form of paid work, either as an employee or as self-employed. Unemployed entails receiving either social assistance or unemployment insurance while being available for the labor market or undertaking workfare. Outside the labor force entails receiving public welfare without work or workfare requirements. We also obtain the same information for patients' spouses. The information is obtained at the end of November for each year.

Counterfactual Outcomes

First, let $\underline{z}$ be the lowest observed values of instrument (the contact ratio where the likelihood of admission is lowest) and $\bar{z}$ denote the highest observed values of the instrument (the contact ratio where the likelihood of admission is highest). Define the share of always-takers π_a as $p(D = 1|z = \underline{z})$ and the share of never-takers π_n $p(D = 0|z = \bar{z})$. From monotonicity it follows that the share of compliers $\pi_c = 1 - \pi_a - \pi_n$. The outcomes of those who are admitted at the lowest likelihood of admission $\underline{z}$ are a weighted average of always-takers' and compliers' outcomes (Y_1). Likewise, the outcomes of those who are not admitted at highest likelihood of admission $\bar{z}$ are a weighted average of never-takers and compliers (Y_0). We can rearrange this to give us:

$$\begin{aligned} E(Y_1|D_{\underline{z}} = 1, D_{\bar{z}} = 0) &= \frac{\pi_a + \pi_c}{\pi_c} E(Y|D = 1, z = \underline{z}) - \frac{\pi_a}{\pi_c} E(Y|D = 1, z = \bar{z}) \\ E(Y_0|D_{\underline{z}} = 1, D_{\bar{z}} = 0) &= \frac{\pi_n + \pi_c}{\pi_c} E(Y|D = 0, z = \bar{z}) - \frac{\pi_n}{\pi_c} E(Y|D = 0, z = \underline{z}) \end{aligned} \tag{1}$$

We observe all of the moments on the RHS in equation (1) directly in the data.

B Supplementary Figures, Tables and Estimation Results

Table B.1: Balancing test: Instruments against GP Visits, Specialists Visits, Hospital Selection

	Model 1	Model 2	Model 3
A: GP visits, year of admission			
IV, week prior to contact	0.818	0.359	0.360
	(1.885)	(1.222)	(1.240)
B: GP visits, year prior to admission			
IV, week prior to contact	0.170	-0.040	-0.050
	(1.078)	(0.601)	(0.628)
C: Specialist practitioner visits, year of admission			
IV, week prior to contact	0.239	0.132	0.127
	(0.214)	(0.212)	(0.213)
D: Specialist practitioner visits, year prior to admission			
IV, week prior to contact	-0.462	-0.303	-0.299
	(0.351)	(0.277)	(0.303)
E: Contacted county's default hospital			
IV, week prior to contact	-0.117	-0.112	-0.112
	(0.143)	(0.124)	(0.124)
F: Contacted county's default hospital (excl. Copenhagen)			
IV, week prior to contact^{A}	-0.076	0.066	-0.067
	(0.166)	(0.141)	(0.142)
G: Contacted county's default hospital (Actual IV relative to default local hospitals')			
ΔIV, week prior to contact	0.088	0.141	0.143
	(0.111)	(0.108)	(0.107)
SES controls		X	X
Diagnosis controls			X
N	24,277	24,277	24,277

$^{+}p < .10$;$^{*}p < .05$;$^{**}p < .01$; $^{***}p < .001$

Note: Table shows OLS regression results of the intrumental variable (hospital specific contact intensity the week prior to the individual's initial contact) on number of visits to general practitioners (panel A-B) and specialist practitioners (panel C-D) the year of and the year prior to initial contact, and whether the patient contacted his/her default hospital or not (panel E-F). Panel G shows regression of difference in contact intensity between the actual contacted hospital and the default hospital on whether the patient contacted his/her default hospital or not. Standard errors clustered by hospital and month in parentheses. SES and demographic controls include: Gender (dummy), age at adm., mother's age at birth, mothers years of schooling, father' age at birth, father's years of schooling, mother has prior psych. history (dummy), admitted in own municipality (dummy), greater CPH area (dummy), other metropolitan area (dummy), year dummies. Diagnosis controls include: Dummies for each F. diagnosis category from ICD-10.

^{A} : N excl. Copenhagen = 19595.

Source: Own calculations on data from Statistics Denmark.

Table B.2: Balancing test: Instrument against Covariates

	IV
Male	0.005
	(0.003)
Age at admission	0.000
	(0.000)
Mother's age at birth	-0.000
	(0.000)
Mother's months of schooling	-0.000
	(0.000)
Year 1999	0.022
	(0.038)
Year 2000	-0.014
	(0.035)
Mother been admitted to psychiatric hospital	-0.000
	(0.003)
Mother missing	-0.002
	(0.005)
Father's months of schooling	-0.000
	(0.000)
Father missing	-0.009
	(0.006)
Admitted in own municipality	0.013
	(0.014)
Admitted in Copenhagen	-0.041
	(0.038)
Admitted in metropolitan area	-0.071*
	(0.033)
Admitted in Funen or Jutland	0.040
	(0.044)
Disorder associated with substance use	0.002
	(0.012)
Psychosis, schizophrenia	0.003
	(0.010)
Anxiety or stress-related	0.004
	(0.009)
Personality disorder	0.007
	(0.009)
Affective/emotional, pre-adult origin	-0.001
	(0.015)
Previous crime (0/1)	0.001
	(0.004)
Out of labor force in year -1	0.002
	(0.003)
Unemployed in year -1	-0.001
	(0.005)
Municipality mean, out of labor force	0.041
	(0.059)
Municipality mean, unemployed	0.021
	(0.070)
Observations	24,277

$^{+}p < .10; ^{*}p < .05; ^{**}p < .01; ^{***}p < .001$

Note: Table shows OLS regression results of the intrumental variable (hospital specific contact intensity the week prior to the individual's initial contact) on gender (dummy), age at adm., mother's age at birth, mothers years of schooling, father' age at birth, father's years of schooling, mother has prior psych. history (dummy), admitted in own municipality (dummy), greater CPH area (dummy), other metropolitan area (dummy), in regions Funen or Jutland (dummy), year dummies, and dummies for each F. diagnosis category form ICD-10. Standard errors clustered by hospital in parentheses.

Source: Own calculations on data from Statistics Denmark.

Table B.3: 1st stage Estimation Results

Subgroup:	Females	Males	Age $\leq$ 30 before contact	Age > 30 before contact	No crime before contact	Crime before contact
Week prior to contact	0.150*	0.193*	0.155*	0.187*	0.172*	0.170*
	(0.055)	(0.060)	(0.054)	(0.059)	(0.053)	(0.071)
F-value	7.39	10.50	8.19	9.92	10.62	5.80
Observations	13,031	11,246	12,071	12,206	17,486	6,791
SES and demographic controls	X	X	X	X	X	X
Diagnosis controls	X	X	X	X	X	X

$^{+}p < .10; ^{*}p < .05; ^{**}p < .01; ^{***}p < .001$

Note: Table shows OLS regression results of hospital admission (0/1) on the instrumental variable; hospital specific contact intensity the week prior to the individual's initial contact for different subgroups in the sample. Standard errors clustered by hospital and month in parentheses. SES and demographic controls include (each specific subgroup variable excluded): Gender (dummy), age at adm., mother's age at birth, mothers years of schooling, father' age at birth, father's years of schooling, mother has prior psych. history (dummy), admitted in own municipality (dummy), greater CPH area (dummy), other metropolitan area (dummy) (CPH and metropolitan dummies are excluded when including region FEs), year dummies. Diagnosis controls include: Dummies for each F. diagnosis category from ICD-10.

Source: Own calculations on data from Statistics Denmark.

Table B.4: Reduced form estimates for crime

	Crime, 3m period	Crime, Accum
3 months from first contact	-0.009	-0.009
	(0.006)	(0.006)
6 months from first contact	-0.006	-0.016^{+} 4
	(0.005)	(0.008)
12 months from first contact	-0.001	-0.018
	(0.007)	(0.015)
24 months from first contact	-0.000	-0.034
	(0.006)	(0.021)
36 months from first contact	-0.001	-0.024
	(0.006)	(0.029)
Observations	24,277	24,277
SES and demographic controls	X	X
Diagnosis controls	X	X

$^{+}p < .10$;$^{*}p < .05$;$^{**}p < .01$; $^{***}p < .001$

Note: Table shows reduced form results of the instrument on crimes. Time 0 is month of initial contact. Panel A shows results for quarterly rates, i.e. from month 1-3, 4-6, 10-12, 22-24, and 34-36. Panel B shows results for rates accumulated from time of first contact. Standard errors clustered by hospital and month in parentheses.

SES and demographic controls include: Gender (dummy), age at adm., mother's age at birth, mothers years of schooling, father' age at birth, father's years of schooling, mother has prior psych. history (dummy), admitted in own municipality (dummy), greater CPH area (dummy), in regions Funen or Jutland (dummy), other metropolitan area (dummy), year dummies. Diagnosis controls include: Dummies for each F. diagnosis category from ICD-10.

Source: Own calculations on data from Statistics Denmark.

Table B.5: Reduced form estimates of admission on subsequent contact and admission to psychiatric hospitals and use of GP and specialist practitioner

A: New contact	
1 week from first contact	-0.034*
	(0.014)
1 week from first contact, to new hospital	0.005
	(0.008)
3 months from first contact	0.037
	(0.034)
6 months from first contact	0.007
	(0.022)
12 months from first contact	-0.004
	(0.009)
B: New admission	
1 week from first contact	0.007
	(0.010)
3 months from first contact	0.036+
	(0.019)
6 months from first contact	0.004
	(0.014)
12 months from first contact 0.004	
	(0.006)
C: Use of GP or specialist practitioner	
GPs, year 1 from first contact	-0.425
	(0.280)
Specialists, year 1 from first contact	-0.111*
	(0.052)
GPs, year 2 from first contact	-0.230
	(0.211)
Specialists, year 2 from first contact	-0.041
	(0.031)
Observations	24,277
SES and demographic controls	X
Diagnosis controls	X

$^{+}p < .10$;$^{*}p < .05$;$^{**}p < .01$; $^{***}p < .001$

Note: Table shows reduced form estimates of the instrument on probability of subsequently contacting a psychiatric hospital again, the probability of subsequently being admitted to a psychiatric hospital again, and the number of contacts / treatments at GPs or specialist practitioner. Time 0 is month of initial contact. Table shows results for quarterly contact and admission rates, i.e. from month 0-3, 3-6, 9-12, and for GPs and specialists the first two years following first contact to a psychiatric hospital. Standard errors clustered by hospital in parentheses.

SES and demographic controls include: Gender (dummy), age at adm., mother's age at birth, mothers years of schooling, father' age at birth, father's years of schooling, mother has prior psych. history (dummy), admitted in own municipality (dummy), greater CPH area (dummy), in regions Funen or Jutland (dummy), other metropolitan area (dummy), year dummies. Diagnosis controls include: Dummies for each F. diagnosis category from ICD-10.

Source: Own calculations on data from Statistics Denmark.

Table B.6: Estimation Results, Heterogeneity by Observable Characteristics

	Reduced form estimates						IV estimates					
Subgroup:	Males	Females	Age ≤ 30	Age > 30	No crime before contact	Crime before contact	Males	Females	Age ≤ 30	Age > 30	No crime before contact	Crime before contact
Panel A: Crimes												
3 months	-0.026*	0.005	-0.021*	0.002	0.003	-0.041*	-0.113*	0.031	-0.117**	0.008	0.015	-0.203+
	(0.010)	(0.004)	(0.008)	(0.008)	(0.003)	(0.018)	(0.056)	(0.028)	(0.045)	(0.036)	(0.017)	(0.106)
6 months	-0.046**	0.011*	-0.031*	-0.001	0.005	-0.071*	-0.203+	0.062+	-0.172*	-0.007	0.026	-0.353+
	(0.016)	(0.004)	(0.014)	(0.015)	(0.007)	(0.029)	(0.105)	(0.037)	(0.078)	(0.066)	(0.031)	(0.206)
1 year	-0.059+	0.016	-0.040	0.001	0.003	-0.078	-0.261	0.089	-0.224+	0.005	0.017	-0.385
	(0.029)	(0.010)	(0.025)	(0.022)	(0.006)	(0.055)	(0.161)	(0.064)	(0.127)	(0.094)	(0.026)	(0.325)
11 years	-0.290*	0.013	-0.193*	-0.070	0.011	-0.497*	-1.502+	0.088	-1.245+	-0.375	0.063	-2.920+
	(0.114)	(0.033)	(0.089)	(0.083)	(0.024)	(0.185)	(0.790)	(0.217)	(0.654)	(0.497)	(0.130)	(1.748)
Observations	11,246	13,031	12,071	12,206	17,486	6,791	11,246	13,031	12,071	12,206	17,486	6,791
SES and demogr. controls	X	X	X	X	X	X	X	X	X	X	X	X
Diagnosis controls	X	X	X	X	X	X	X	X	X	X	X	X

Note: Table shows reduced form and 2SLS regression results of hospital admission (0/1) on subsequent crimes. Time 0 is month of initial contact. Outcomes are measured as accumulated from time of discharge. Standard errors clustered by hospital in parentheses. SES and demographic controls include: Gender (dummy), age at adm., mother's age at birth, mothers years of schooling, father' age at birth, father's years of schooling, mother has prior psych. history (dummy), admitted in own municipality (dummy), greater CPH area (dummy), other metropolitan area (dummy), in regions Funen or Jutland (dummy), year dummies. Diagnosis controls include: Dummies for each F. diagnosis category from ICD-10. Source: Own calculations on data from Statistics Denmark. $^{+}p < .10; ^{*}p < .05; ^{**}p < .01; ^{***}p < .001$

Table B.7: $2SLS$ and $2SRI$ models for violent crime, property crime, and other crime

	Acc. property crime			*Acc. violent crime*			*Acc. other crime*		
	$2SLS$	$2SRI$	AME	$2SLS$	$2SRI$	AME	$2SLS$	$2SRI$	AME
3 m after contact	-0.041	-2.293	-0.015	-0.012	-0.822	-0.001	-0.021	-9.943*	-0.023
	(0.038)	(1.555)		(0.016)	(3.494)		(0.017)	(4.070)	
6 m after contact	-0.075	-1.933^{+}	-0.025	-0.037	-0.707	-0.002	-0.051^{+}	-8.419**	-0.043
	(0.058)	(1.134)		(0.023)	(2.243)		(0.030)	(2.964)	
N	24,277	24,277		24,277	24,277		24,277	24,277	

$^{+}p < .10$;$^{*}p < .05$;$^{**}p < .01$; $^{***}p < .001$

Note: Standard errors and asymptotically correct standard errors in parentheses. AME: Average marginal effect. Time 0 is month of initial contact. Standard errors clustered by hospital and month in parentheses. SES and demographic controls include: Gender (dummy), age at adm., mother's age at birth, mothers years of schooling, father' age at birth, father's years of schooling, mother has prior psych. history (dummy), admitted in own municipality (dummy), greater CPH area (dummy), other metropolitan area (dummy), year dummies. Other crime includes drugs-related and weapons-related crime. Diagnosis controls include: Dummies for each F. diagnosis category from ICD-10.

Source: Own calculations on data from Statistics Denmark.

Table B.8: Effect of admission on number of crimes, the role of incapacitation

	A: From day of discharge		*B: From day of first contact from Tables 3*	
	AME_{2SRI}	2SLS	AME_{2SRI}	2SLS
A: Number of crimes accumulated from contact				
3 months from first contact	-0.044	-0.043	-0.039*	-0.054^{+}
	(0.029)	(0.057)	(0.018)	(0.030)
6 months from first contact	-0.070	-0.056	-0.068*	-0.090^{+}
	(0.046)	(0.093)	(0.028)	(0.052)
12 months from first contact	-0.110	-0.020	-0.117*	-0.105
	(0.075)	(0.151)	(0.052)	(0.086)
Observations	24,277	24,277	24,277	24,277
SES and demographic controls	X	X	X	X
Diagnosis controls	X	X	X	X

$^{+}p < .10$;$^{*}p < .05$;$^{**}p < .01$; $^{***}p < .001$

Note: Table shows reduced form and 2SLS regression results of hospital admission (0/1) on subsequent crimes. Time 0 is month of discharge. Standard errors clustered by hospital in parentheses.

SES and demographic controls include: Gender (dummy), age at adm., mother's age at birth, mothers years of schooling, father' age at birth, father's years of schooling, mother has prior psych. history (dummy), admitted in own municipality (dummy), greater CPH area (dummy), other metropolitan area (dummy), in regions Funen or Jutland (dummy), year dummies. Diagnosis controls include: Dummies for each F. diagnosis category from ICD-10.

Source: Own calculations on data from Statistics Denmark.

Table B.9: Effect of admission on labor earnings, extensive and intensive margin effects

	Reduced form estimates			2SLS estimates		
Labor earnings, \$1,000	0	1-30	>30	0	1-30	>30
A: Employment						
12 months from first contact	0.006	0.016	-0.023	0.038	0.092	-0.135
	(0.025)	(0.014)	(0.022)	(0.142)	(0.084)	(0.125)
24 months from first contact	0.019	0.018*	-0.035^{+}	0.113	0.108^{+}	-0.206^{+}
	(0.017)	(0.009)	(0.018)	(0.096)	(0.060)	(0.116)
36 months from first contact	0.025	0.020	-0.045**	0.144	0.119	-0.261*
	(0.016)	(0.013)	(0.016)	(0.094)	(0.077)	(0.105)
Observations	24,277	24,277	24,277	24,277	24,277	24277
SES and demographic controls	X	X	X	X	X	X
Diagnosis controls	X	X	X	X	X	X

$^{+}p < .10$;$^{*}p < .05$;$^{**}p < .01$; $^{***}p < .001$

Note: Table shows reduced form and 2SLS regression results of hospital admission (0/1) in the probability of having labor earnings within three intervals: 0 (corresponding to not being employed), \$1,000-30,000 (suggesting part time work), and >\$30,000 (suggesting full time work). Time 0 is month of initial contact. Standard errors clustered by hospital in parentheses.

SES and demographic controls include: Gender (dummy), age at adm., mother's age at birth, mothers years of schooling, father' age at birth, father's years of schooling, mother has prior psych. history (dummy), admitted in own municipality (dummy), greater CPH area (dummy), other metropolitan area (dummy), in regions Funen or Jutland (dummy), year dummies. Diagnosis controls include: Dummies for each F. diagnosis category from ICD-10.

Source: Own calculations on data from Statistics Denmark.

Figure B.1: Distribution of instrumental variable

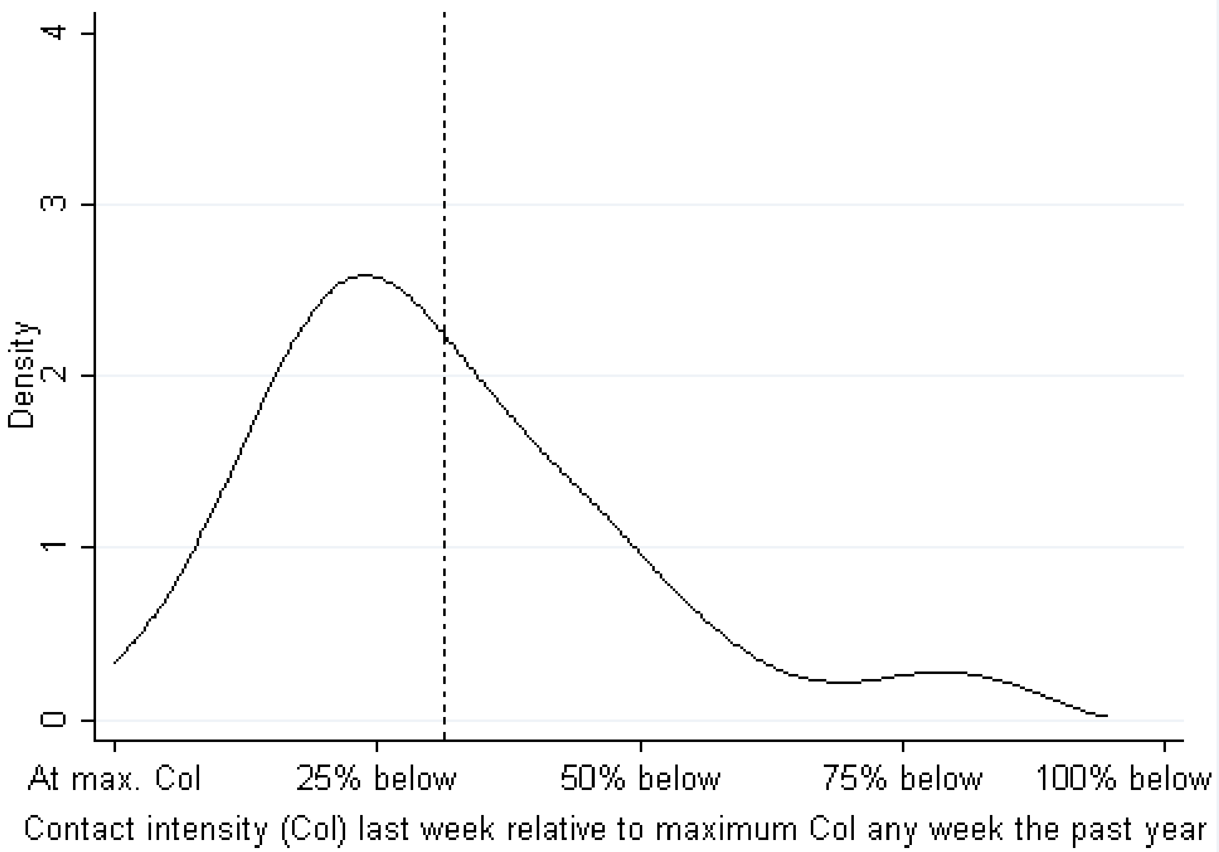

Note: Dashed line indicates mean.
Source: Own calculations on data from Statistics Denmark.

Figure B.2: Potential labor market outcomes for compliers

(a) Employment

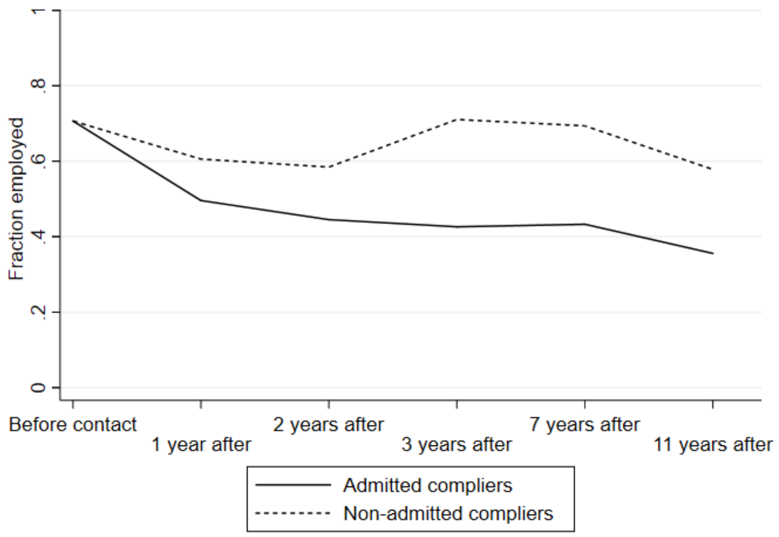

(b) Unemployment

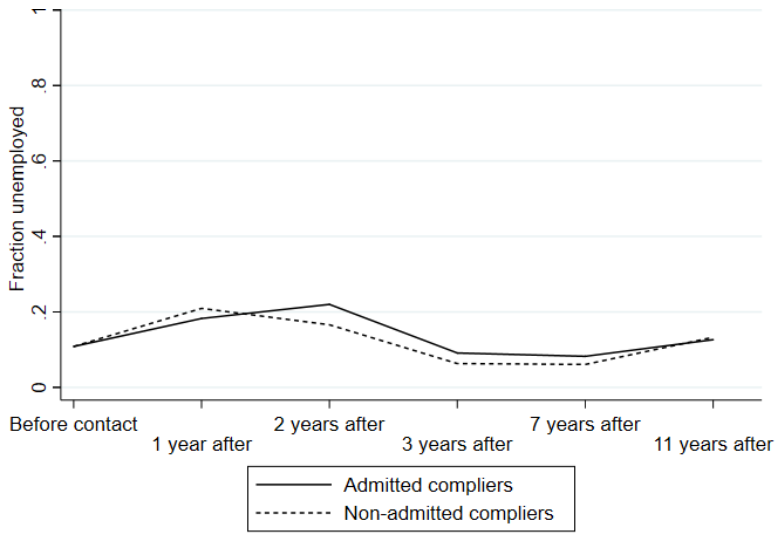

(c) Not in labor force

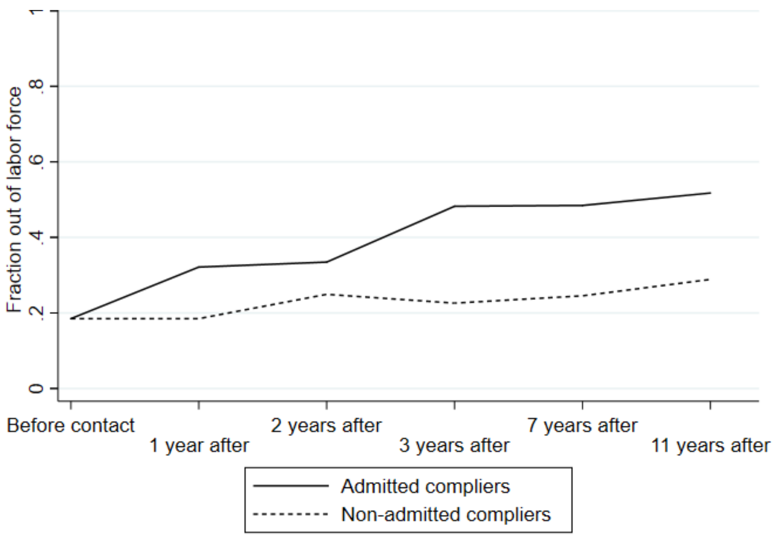

Note: Figures show estimated potential outcomes for compliers the year before and three years after first contact to a psychiatric hospital following Dahl et al. (2014), Appendix B. Potential outcomes are estimated using both instruments (hospital specific contact intensity the weeks prior to contact) but not any covariates.
Source: Own calculations on data from Statistics Denmark.

References

Dahl, G. B., A. R. Kostøl, and M. Mogstad (2014). Family welfare cultures. *The Quarterly Journal of Economics 129*(4), 1711–1752.

WHO (2011). *Mental Health Atlas.* Geneva, CH: WHO.
